# Opposite Root Growth Phenotypes of *hy5* versus *hy5 hyh* Mutants Correlate with Increased Constitutive Auxin Signaling

**DOI:** 10.1371/journal.pgen.0020202

**Published:** 2006-11-24

**Authors:** Richard Sibout, Poornima Sukumar, Chamari Hettiarachchi, Magnus Holm, Gloria K Muday, Christian S Hardtke

**Affiliations:** 1 Department of Plant Molecular Biology, University of Lausanne, Lausanne, Switzerland; 2 Department of Biology, Wake Forest University, Winston-Salem, North Carolina, United States of America; 3 Department of Cell and Molecular Biology, Gothenburg University, Gothenburg, Sweden; University of Wisconsin, United States of America

## Abstract

The *Arabidopsis* transcription factor HY5 controls light-induced gene expression downstream of photoreceptors and plays an important role in the switch of seedling shoots from dark-adapted to light-adapted development. In addition, HY5 has been implicated in plant hormone signaling, accounting for the accelerated root system growth phenotype of *hy5* mutants. Mutants in the close *HY5* homolog *HYH* resemble wild-type, despite the largely similar expression patterns and levels of *HY5* and *HYH,* and the functional equivalence of the respective proteins. Moreover, the relative contribution of *HYH* to the overall activity of the gene pair is increased by an alternative *HYH* transcript, which encodes a stabilized protein. Consistent with the enhanced root system growth observed in *hy5* loss-of-function mutants, constitutively overexpressed alternative HYH inhibits root system growth. Paradoxically, however, in double mutants carrying *hy5* and *hyh* null alleles, the *hy5* root growth phenotype is suppressed rather than enhanced. Even more surprisingly, compared to wild-type, root system growth is diminished in *hy5 hyh* double mutants. In addition, the double mutants display novel shoot phenotypes that are absent from either single mutant. These include cotyledon fusions and defective vasculature, which are typical for mutants in genes involved in the transcriptional response to the plant hormone auxin. Indeed, many auxin-responsive and auxin signaling genes are misexpressed in *hy5* mutants, and at a higher number and magnitude in *hy5 hyh* mutants. Therefore, auxin-induced transcription is constitutively activated at different levels in the two mutant backgrounds. Our data support the hypothesis that the opposite root system phenotypes of *hy5* single and *hy5 hyh* double mutants represent the morphological response to a quantitative gradient in the same molecular process, that is gradually increased constitutive auxin signaling. The data also suggest that *HY5* and *HYH* are important negative regulators of auxin signaling amplitude in embryogenesis and seedling development.

## Synopsis

Genetic redundancy is the total or partial compensation of inactivation of one gene by another, usually related gene. In *Arabidopsis, HY5* and *HYH* are highly similar, principally exchangeable genes. However, only inactivation of *HY5* results in morphological defects, indicating that *HY5* plays a more important role in development than *HYH*. Nevertheless, if inactive versions of such partially redundant genes are combined in a double mutant, the defects observed in the single mutant often worsen. Paradoxically, however, combined inactivation of *HY5* and *HYH* leads to a defect that is opposite to inactivation of *HY5* alone: compared to controls, root system growth is decreased in the double mutant, rather than enhanced as in plants only lacking *HY5* activity. Through careful analysis of the double mutant defects and scans of genome-wide gene expression levels, the authors determined that the opposite root system growth of *hy5* single and *hy5 hyh* double mutants is a morphological response to a gradually increased quantitative disturbance in the same molecular process, the physiological response to the plant hormone auxin. This example suggests that inactivation of genes that quantitatively affect the balance of a physiological process in the same manner might manifest in very different morphological changes.

## Introduction

Homologous genes of the same family display genetic redundancy to varying degrees if their expression pattern and their function overlap. In general, loss-of-function mutations of redundantly acting genes are expected to result in no phenotype in the case of full redundancy, or similar phenotypes in the case of partial redundancy. If the mutations in partially redundant genes are combined, an enhancement of the single mutant phenotypes is expected. In this study, we investigated the genetic redundancy between two functionally equivalent *Arabidopsis* transcription factors. Surprisingly, their combined loss-of-function leads to a phenotype that is opposite to what would be expected from the single mutant phenotypes. These two genes have been originally identified because of their role in light signaling.

Light is arguably the most important stimulus in plant development, since growth and reproductive success ultimately depend on the energy harvested from light by photosynthesis. To sense the intensity, direction, and spectral quality of light, plants have developed sophisticated molecular networks [1]. Plants also possess circadian clocks to measure day length and to adjust their physiology in anticipation of dawn [2]. Within the light-sensing network, a few factors have a central role in the downstream transcriptional response. Their importance is particularly evident in the most extreme light environment transition in the plant life cycle, the transition from dark-adapted (skotomorphogenic) to light-adapted (photomorphogenic) development.

Skotomorphogenic seedlings display closed cotyledons, which protect the shoot meristem, reduced root growth, and strongly enhanced hypocotyl elongation. By this behavior, seedlings concentrate their resources toward pushing the shoot meristem through the soil into the light in nature. Light exposure then triggers photomorphogenesis, which comprises light-induced gene expression, cotyledon expansion, photosynthesis, suppression of hypocotyl elongation, and acceleration of root and shoot growth. Factors involved in the transition from skotomorphogenesis to photomorphogenesis have mainly been identified in *Arabidopsis*. Among them, the basic leucine zipper (bZIP) transcription factors LONG HYPOCOTYL 5 (HY5) and HY5 HOMOLOG (HYH) play an important role in light-induced gene expression. Loss-of-function *hy5* mutants display dark-grown characteristics in the light [3], most significantly, a loss of the inhibition of hypocotyl elongation. While *hy5* mutants display this phenotype in all light conditions, mutants in *hyh* show a similar but very weak phenotype only in blue light [4].

A general characteristic of the transition from skotomorphogenesis to photomorphogenesis is the suppression of cell expansion in some organs, for instance, the hypocotyl, increased cell expansion in others, e.g., the cotyledons, and the onset of growth by cell division in the shoot and root meristems. Notably, both cell expansion and division are thought to be under crucial control of plant hormone signals. Thus, it has long been suspected that light signaling must intersect with hormone signaling or biosynthesis pathways to elicit the desired responses. In fact, several plant hormones have been implicated in light signaling, because they influence cell expansion and/or division, or light-regulated gene expression [5–9]. Among them, auxin is of particular interest, because it is known to regulate cell elongation as well as division in a dosage-dependent fashion. Accordingly, several genes that act within the auxin signaling framework have been implicated in light responses [10–14].

*HY5* has also been implicated in auxin signaling, partly based on photomorphogenic traits of *hy5* mutants [3,15] but mainly because of the *hy5* root system phenotypes. The *Arabidopsis* root system is dominated by a primary root, which is formed during embryogenesis. After germination, this primary root grows, driven by the cell proliferation and elongation taking place in its apical meristem. Once the primary root has reached a certain length several days after germination, the root system extends through the formation of lateral roots, which emerge along the primary root. In *hy5* mutants, the emergence of lateral roots occurs earlier than in wild-type, resulting in overall enhanced root system growth [3,15]. Moreover, the gravitropism of *hy5* roots is reduced. Both lateral root formation and root gravitropism are known to require an intact auxin signaling pathway.

Here we present a detailed analysis of the genetic redundancy between *HY5* and its homolog, *HYH,* which revealed paradoxically opposite root system phenotypes of *hy5* single versus *hy5 hyh* double mutants. These phenotypes correlate with quantitatively different constitutive auxin signaling in the mutants. Our data suggest that both genes act redundantly as quantitative modulators of auxin signaling and have a much more central role in this process than anticipated from their respective single mutant phenotypes. This role goes far beyond their role in light signaling and impinges on embryogenesis, root development, shoot development, and vascular differentiation.

## Results

### The Expression Patterns of *HY5* and *HYH* Are Largely Similar

**Figure 1:**
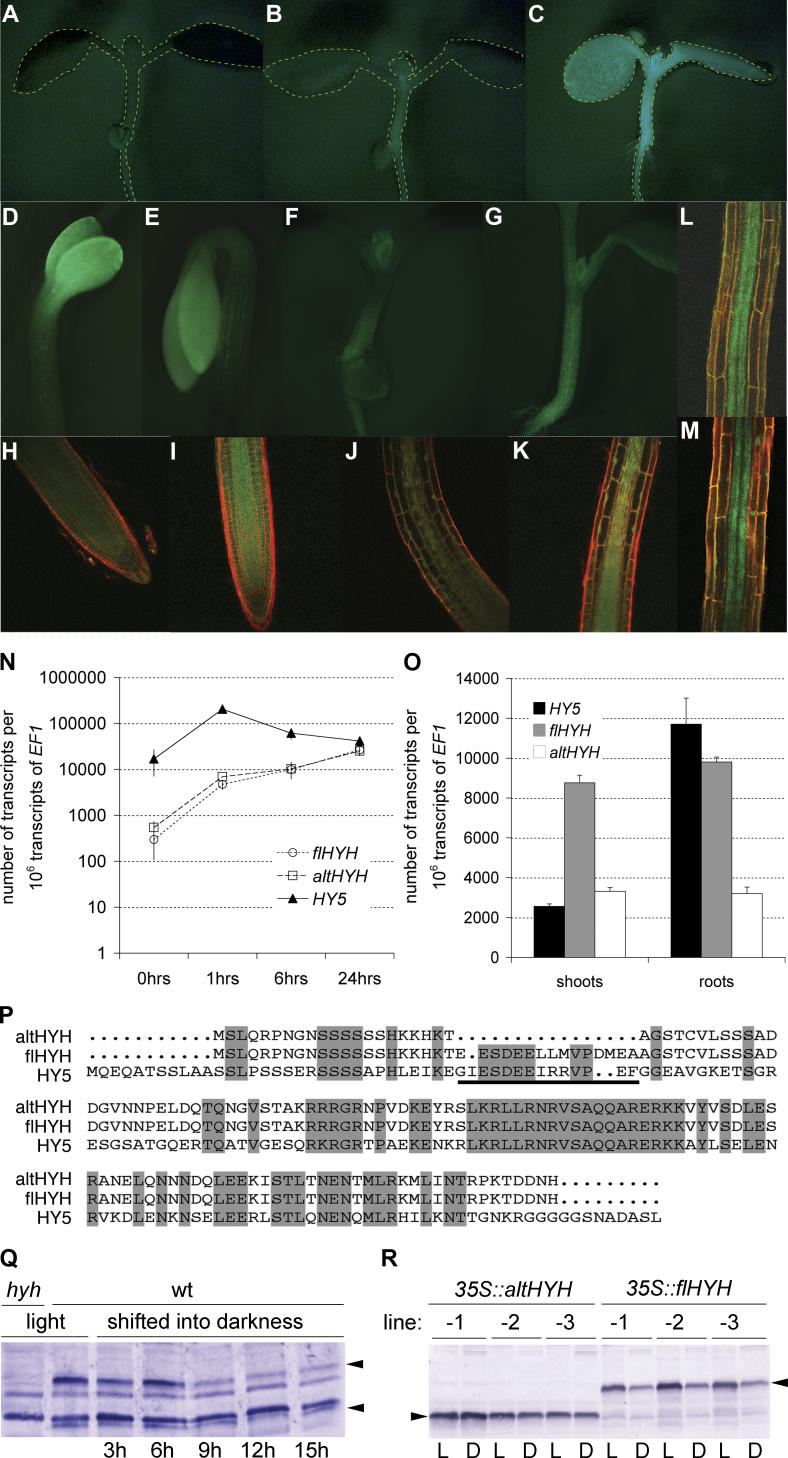
Expression Analysis of *HY5* and *HYH* Transcripts and Proteins (A–M) Analysis of transgenic wild-type plants expressing the GFP reporter (green fluorescence) under control of the *HY5* (B, D, F, H, J, L) or *HYH* (C, E, G, I, K, M) promoter. Seedling shape is indicated by a broken line in (A–C) for clarity. (A) A light-grown wild-type control seedling. (B and C) Light-grown seedlings at 5 d after germination. (D and E) Apical part of the hypocotyl of dark-grown seedlings, including cotyledons. (F and G) Close-up of the hypocotyl of light-grown seedlings. (H and I) Primary root tip. (J and K) Elongation zone of the primary root meristem. (L and M) Mature part of the primary root. Images (A–D) have been acquired by fluorescence microscopy, (E–J) by confocal microscopy. (N) qPCR quantification of *HY5* and *HYH* transcripts in dark-grown seedlings (t = 0) shifted into the light (intensity 5 μE), assayed at given time points. Transcript quantity is expressed in relation to the control gene *EF1*. (O) Abundance of *HY5* and *HYH* transcripts in shoots or roots of 6-d-old seedlings measured by qPCR. Seedlings were grown in constant light at an intensity of 100 μE. Transcript quantity is expressed in relation to the control gene *EF1*. (P) Alignment of conceptually translated ORFs of *HY5* and *HYH* cDNAs. The COP1 interaction domain is underlined. altHYH indicates protein derived from alternatively spliced *HYH* transcript; flHYH, protein derived from full-length *HYH* transcript. (Q) Western analysis of dark-induced degradation of endogenous HYH proteins. Seven-day-old light-grown seedlings were shifted into darkness and samples were removed at the indicated time points. The two HYH isoforms detected by anti-HYH antibody are indicated by arrowheads. Cross-hybridizing bands serve as loading control and can be identified by comparison with the extract from *hyh* null mutants. Expected protein sizes are 15.2 and 16.9 kDa, and observed sizes are 18 and 25 kDa for altHYH and flHYH, respectively. The discrepancy is likely due to the highly charge of the proteins [4]. (R) Western analysis of dark-induced degradation of transgenic HYH proteins. Six-day-old light-grown seedlings expressing either *flHYH* or *altHYH* under control of the *35S* promoter were kept in the dark for 24 h and compared to controls that were kept in the light. The two HYH isoforms detected by anti-HYH antibody are indicated by arrowheads. Endogenous HYH proteins (see N) are not detected because the transgenes were expressed in a *hy5 hyh* background. Error bars represent standard error of the mean.

In contrast to the pronounced seedling shoot phenotypes of *hy5* null mutants, *hyh* null mutants display only very weak phenotypes and only so in blue light. These are slightly decreased anthocyanin biosynthesis and slight decreased inhibition of hypocotyl elongation [4]. In darkness and white light, *hyh* mutants resemble wild-type. The discrepancy between the *hy5* and *hyh* mutant phenotypes could be explained by differential expression patterns of the two genes. To test this hypothesis, we constructed transgenic plants expressing a *GFP* reporter gene under control of either the *HY5* or the *HYH* promoter. Similar expression patterns for each construct were observed in several independent transgenic lines (Figure 1). In several-day-old light-grown seedlings, *HY5* is expressed mainly in the hypocotyl and only very weakly in the cotyledons (Figure 1B and 1F). At this stage, *HYH* is expressed throughout the seedling as well, but at a clearly higher level than *HY5* (Figure 1C and 1G). In dark-grown seedlings, both genes are expressed in the apical region of the hypocotyl and in the cotyledons (Figure 1D and 1E). Both genes are also strongly expressed throughout the proliferation zone of the root meristem (Figure 1H and 1I). In the elongation zone, expression becomes gradually concentrated in the vasculature (Figure 1J and 1K), where both genes are primarily expressed in the mature root (Figure 1L and 1M). Thus, in general, the expression patterns of *HY5* and *HYH* are largely identical.

### Expression Dynamics of *HY5* and *HYH*

Differences in expression level rather than pattern could provide an alternative explanation for the different importance of the two genes. For instance, the absence of phenotypes in *hyh* mutants could mean that *HYH* is expressed at much lower levels than *HY5*. However, the GFP fluorescence in the *HYH* reporter lines rather appeared to be consistently higher than in the *HY5* lines in light-grown conditions, where the *hy5* phenotypes manifest. To confirm the quantitative difference proposed by the reporter genes, we determined endogenous *HY5* and *HYH* transcript abundance by quantitative real-time RT-PCR (qPCR) in seedlings. Because we detected a truncated alternative *HYH* transcript *(altHYH)* in pilot RT-PCR experiments, the qPCR experiments were designed to differentiate between *altHYH* and full-length *HYH (flHYH).*

In the dark, *HYH* transcripts are present at very low levels, while *HY5* is roughly ten times more abundant (Figure 1N). Upon light stimulus, *HY5* expression is strongly induced 10- to 12-fold within 1 h, before dropping back to approximately twice its dark level within 6 h (Figure 1N). The expression of both *HYH* transcripts is also highly light inducible (approximately 50- to 100-fold; Figure 1N) but at a slower pace. Upon light stimulus, *HYH* levels increase steadily, eventually reaching levels comparable to the later steady state levels of *HY5*. However, the two *HYH* transcripts in combination are about twice as abundant as *HY5* in light-grown seedlings (Figure 1N and 1O). Examination of dissected light-grown shoots and roots reveals that the excess of *HYH* as compared to *HY5* transcript is restricted to the shoot (Figure 1O), with a ratio of *HYH* to *HY5* transcripts of roughly 6-fold. By contrast, in roots the abundance of *HY5* transcript is approximately equal to the abundance of the combined *HYH* transcripts. Thus, in the conditions where *hy5* phenotypes are evident, *HYH* levels are similar to or higher than *HY5* levels.

### altHYH Is Less Susceptible to Proteasome-Mediated Degradation

Another explanation for the differential activity of the two genes could be differential activity of the respective proteins. In this context, altHYH is of particular interest, because in the *altHYH* transcript the coding region for the COP1-interaction domain [16] is spliced out (Figure 1P). CONSTITUTIVE PHOTOMORPHOGENIC 1 (COP1) is a ubiquitin ligase, which targets both HY5 and HYH for proteasome-mediated degradation in the dark [4,17,18]. This process requires the presence of the COP1-interaction domain [16]. Thus, altHYH should not be susceptible to COP1-mediated proteasomal degradation. Anti-HYH antibodies detect a protein, previously interpreted to be an flHYH degradation product [4], which unlike flHYH is not degraded when seedlings are shifted from light to darkness (Figure 1Q). Just like flHYH, this band is missing in *hyh* mutants. To confirm that it represents altHYH, we constructed transgenic plants constitutively overexpressing *altHYH* or *flHYH* ORFs under control of the cauliflower mosaic virus *35S* promoter. The transgenic proteins are detected at similar molecular weight as the endogenous ones. Transgenic altHYH is also not susceptible to degradation in darkness (Figure 1R), suggesting that the corresponding endogenous band is indeed altHYH.

**Figure 2:**
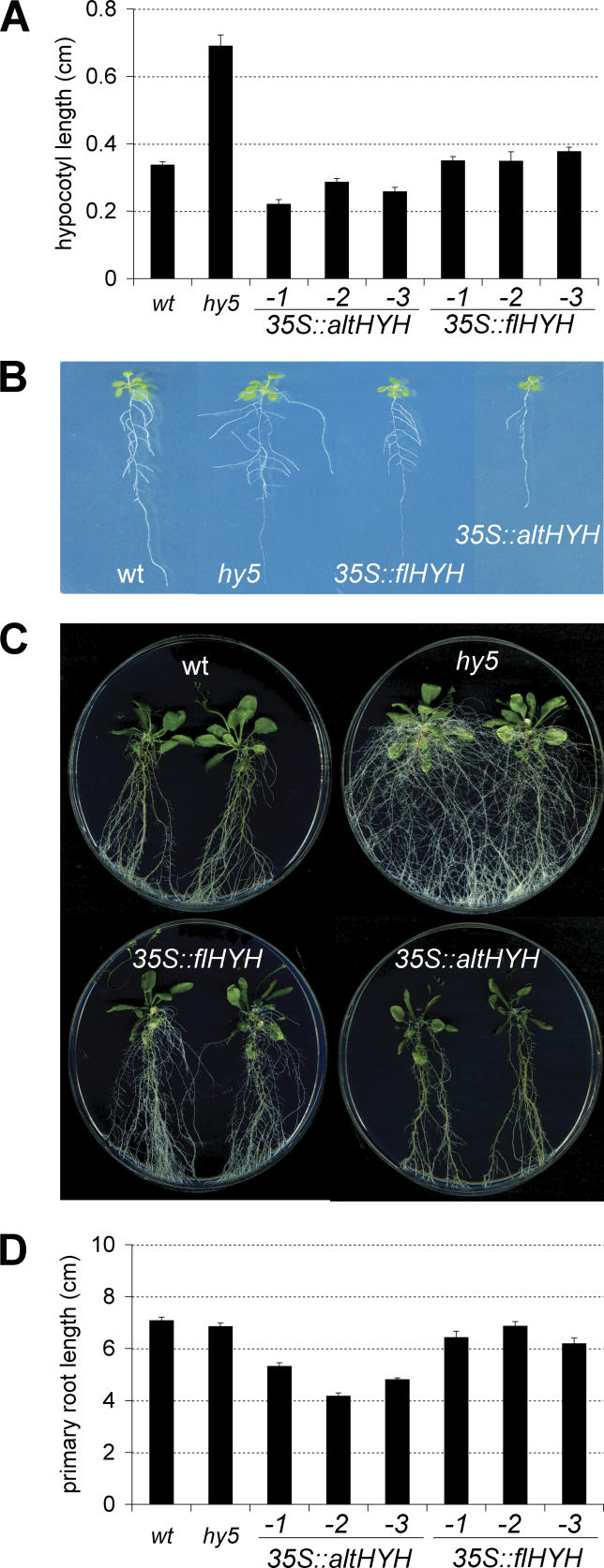
Complementation of *hy5* Phenotypes by *HYH* Transcripts Analyses of independent transgenic lines constitutively overexpressing *altHYH* or *flHYH* under control of the *35S* promoter in a *hy5* background. (A) Hypocotyl length. (B) Lateral root phenotypes of representative plantlets. (C) Root greening phenotypes of representative plants. (D) Primary root length. Plant age: (A and D) = 8 dag (days after germination); (B) = 13 dag; (C) = 30 dag. Error bars represent standard error of the mean.

We also introduced the transgenes into a *hy5* background and assayed their capability to rescue *hy5* phenotypes. Consistent with previous reports [4,19], *35S:flHYH* fully compensates the lack of *HY5* in photomorphogenesis, similar to a *35S:HY5* control construct. The *35S:flHYH* transgene also normalizes the *hy5* root system phenotypes, indicating that *HYH* can in principle replace *HY5* in all aspects of seedling development. *35S:altHYH* also complements *hy5* phenotypes, but even beyond wild-type levels: hypocotyl elongation is more strongly suppressed than in wild-type (Figure 2A), as is lateral root emergence (Figure 2B), while root greening is exaggerated (Figure 2C). Moreover, *hy5* seedlings complemented by *altHYH* also display slightly but significantly reduced primary root growth (Figure 2D). Thus, the results indicate that HY5 and HYH proteins can act functionally equivalent in both shoot and root development. Because the transgenic plants were assayed in constant light conditions, it also appears that *altHYH* is more active than *flHYH*.

### Root System Growth Is Decreased in *hy5 hyh* Mutants

**Figure 3:**
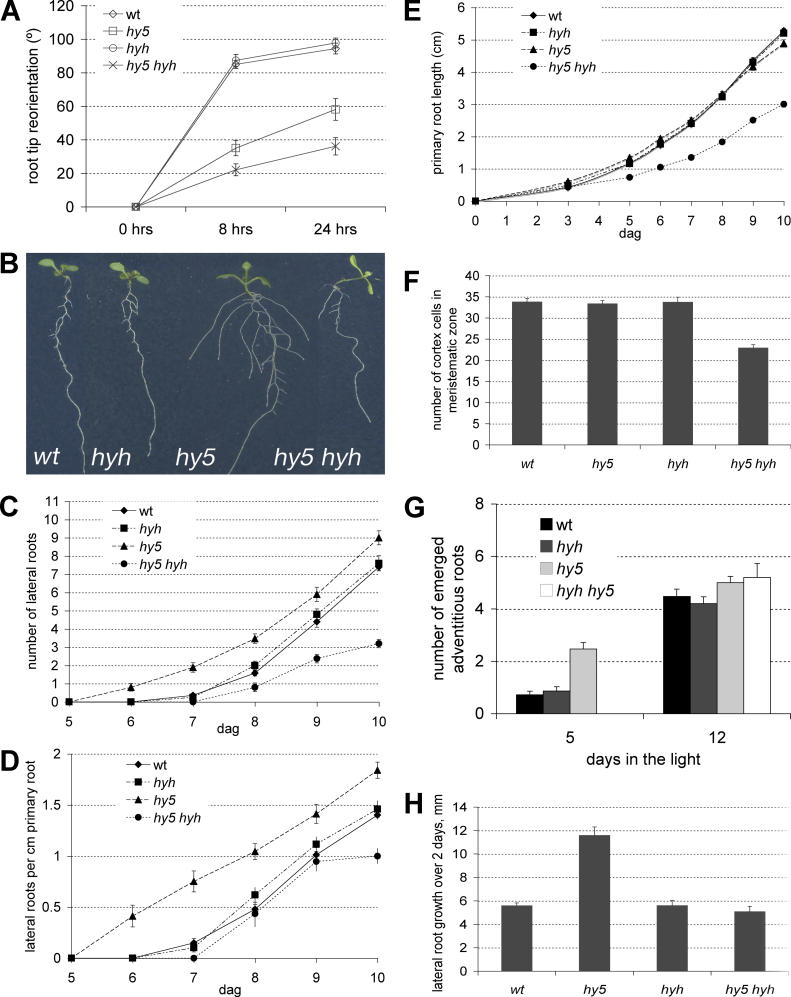
Root System Phenotypes in *hy5 hyh* Double Mutants Phenotypic analyses of wild-type, *hy5*, *hyh,* and *hy5 hyh* seedlings. (A) Gravitropic response of the root tip (curvature). Time points refer to time after change of the gravity vector by 90 degrees. (B) Representative seedlings at 8 dag. (C) Progression of lateral root emergence. (D) Progression of primary root growth of the seedlings in (C). (E) Lateral root density of the seedlings in (C) and (D). (F) Progression of adventitious root formation from the hypocotyl of 5-d-old dark-grown seedlings after shift into the light for the indicated number of days. (G) Lateral root growth rate in the different genotypes, recorded between 8 and 10 dag. (H) Quantification of primary root meristem size. Error bars represent standard error of the mean.

In the two blue light–specific described traits of *hyh*, anthocyanin biosynthesis and hypocotyl elongation, *hy5* and *hyh* behave additively: the relative change conferred by one mutation is proportionally increased by the relative impact of the other mutation [4]. Unlike *hy5* mutants, *hyh* mutants do not display any root system phenotypes. Nevertheless, in *hy5 hyh* double mutants, the agravitropism observed in *hy5* roots is considerably enhanced (Figure 3A). Moreover, the root system growth phenotype of the double mutants represents a reversal of the *hy5* phenotype, because lateral root emergence is delayed rather than enhanced (Figure 3B). Also, the total number of lateral roots formed in the double mutants is reduced (Figure 3C). However, lateral root density is largely similar to wild-type, at least early on (Figure 3D). This is because primary root growth is decreased in the double mutant (Figure 3E). The mature root cell size in the double mutants is similar to the single mutants and wild-type (data not shown), but root meristem size is considerably decreased (Figure 3F). Thus, *hy5 hyh* mutants display reduced root growth because of reduced cell proliferation in the meristem. Delayed formation of lateral organs appears to be a general feature of *hy5 hyh* double mutants, since adventitious root formation on hypocotyls from dark-grown seedlings shifted into light is also delayed (Figure 3G). Finally, the *hyh* mutation not only suppresses the increased lateral root density in *hy5* (Figure 3D) but also suppresses the accelerated growth rate of *hy5* lateral roots (Figure 3H). Thus, in summary, root system growth is enhanced in *hy5* mutants, not affected in *hyh* mutants, but decreased in *hy5 hyh* double mutants.

### Novel Shoot Phenotypes Occur in *hy5 hyh* Mutants

**Figure 4:**
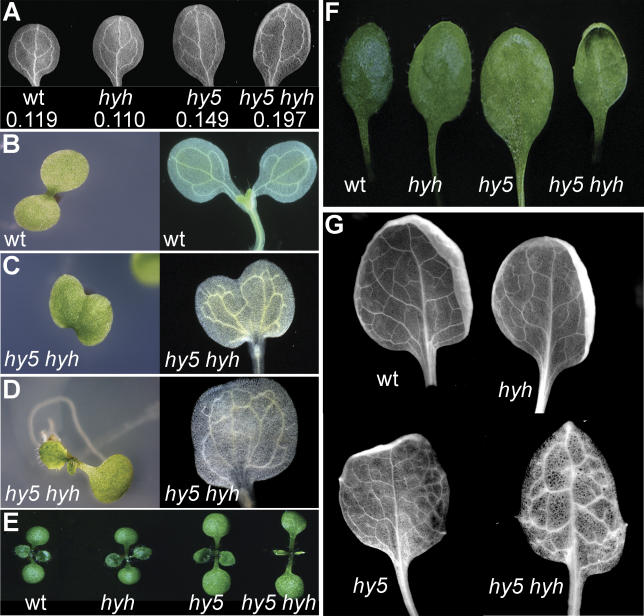
Novel Shoot Phenotypes in *hy5 hyh* Double Mutants Phenotypic analyses of wt, *hy5*, *hyh,* and *hy5 hyh* seedlings. (A) Darkfield microscopy images of cotyledons cleared for visualization of the vasculature. The values for each genotype correspond to the average expanded cotyledon size 7 dag in cm^2^. The respective standard errors of the mean are 0.012, 0.011, 0.019, and 0.023 cm^2^. (B) Wild-type cotyledons, before and after clearing. (C and D) As in (B), for representative fused cotyledons of *hy5 hyh* seedlings. Note the true leaf opposing the fused cotyledon in (D). (E) Representative shoots of 12-d-old light-grown seedlings. (F) Representative first leaves. (G) Darkfield microscopy images of first leaves, cleared for visualization of the vasculature.

In *hy5* shoots, we observed a previously unnoticed increase in cotyledon size (Figure 4). Although cotyledon size is not affected in *hyh,* it is synergistically enhanced in the double mutant (Figure 4A). The double mutant cotyledons also display a novel phenotype that is not found in either single mutant, that is an altered arrangement of the vasculature. Wild-type cotyledon vasculature has a stereotypic pattern, consisting of a midvein and two connected loops on each side of it (Figure 4A). In *hy5*, occasionally three loops or only one loop can be observed. In *hy5 hyh* double mutants, however, the stereotypic arrangement is always broken and most loops are not closed. Moreover, in a considerable proportion (approximately 15%) of double mutant seedlings, the cotyledons are fused, a phenotype never seen in the wild-type or single mutant lines. The degree of fusion is variable and can range from improper separation of cotyledons at their base (Figure 4C) up to total fusion into one big cotyledon (Figure 4D). The vasculature in fused cotyledons is randomly arranged, with incomplete loops and ramifications. In addition, milder phenotypic classes can be observed, such as altered cotyledon arrangement that results in altered phyllotaxis of early leaves.

The alterations in the shoot of *hy5 hyh* seedlings are accompanied by a delayed leaf development (Figure 4E). In particular, the emergence of the first true leaves is significantly delayed, and they display a strong hyponasty (Figure 4F). They also display a strong vascular phenotype, which is again absent from the single mutants. Similar to the cotyledons, the vein pattern is altered, and in addition, the number of strands and ramifications is reduced (Figure 4G). Moreover, the primary veins in the blade run toward the base of the leave instead of away from it. Interestingly, this phenotype is strong in the first four leaves and less penetrant thereafter.

### Transcriptome Alterations in *hy5 hyh* Are More Severe than in *hy5*, but Show the Same Trend

The novel phenotypes observed in *hy5 hyh* double mutants, i.e., reduced root system growth, fused cotyledons, and defective vasculature pattern, are reminiscent of the defects typically observed in many auxin signaling mutants. To determine whether auxin signaling is affected in *hy5 hyh* mutants, we investigated the transcriptome of the double mutants by microarray analyses.

Microarray analyses have been performed previously on *hy5* and *hy5 hyh* mutants [4,15]. However, in these experiments, several-day-old, light-grown seedlings were used. At this stage, the different genotypes display significant morphological differences, which could give rise to expression differences of a secondary nature. To minimize such background and get a grasp on genes primarily affected by *HY5* and *HYH* loss of function, we applied a different strategy. We took into account the finding that both HY5 and HYH protein are very low abundant in dark-grown seedlings [4,17], especially if they have never been exposed to light. Further, there are no morphological differences between dark-grown mutant seedlings and wild-type, except for the occasional cotyledon fusions in the double mutants. Upon exposure to light, *HY5* and *HYH* transcription is induced (see above), and HY5 and flHYH proteins are stabilized within 5 h [4,17], reaching levels comparable to those seen in seedlings grown in constant light. Thus, after 5 h, HY5 and HYH should be fully active. Therefore, for microarray analyses we germinated seedlings in the dark for 3 d and then transferred them into the light. Seedlings were then harvested after 6 h of light exposure and total RNA was isolated and hybridized to Affymetrix ATH1 (http://www.affymetrix.com) microarrays to determine the transcriptome profile for the different genotypes.

**Figure 5:**
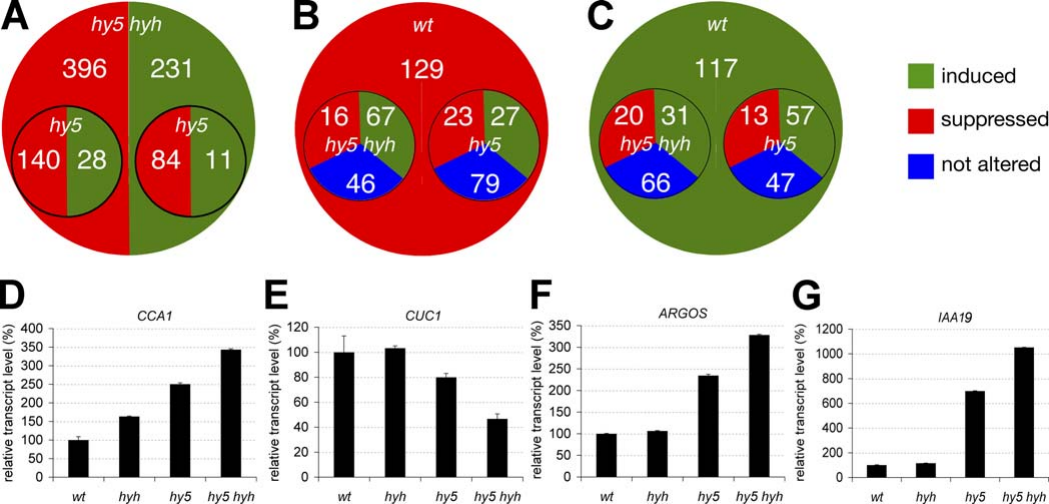
Transcriptome Analysis of *hy5* and *hy5 hyh* Mutants (A) Total number of genes consistently underexpressed or overexpressed in *hy5 hyh* seedlings when compared to wild-type. The inlaid circles represent the subset of genes that are already affected in the *hy5* mutant, with underexpression or overexpression compared to wild-type. (B) Subset of the genes presented in (A) that are repressed by auxin application in wild-type. The inlaid circles represent the auxin response of this set of genes in *hy5* or *hy5 hyh*. (C) Subset of the genes presented in (A) that are induced by auxin application in wild-type. The inlaid circles represent the auxin response of this set of genes in *hy5* or *hy5 hyh*. Red indicates underexpressed in (A) and repressed in (B) and (C). Green indicates overexpressed in (A) and induced in (B) and (C). Blue in (B) and (C) indicates genes that are not auxin responsive any longer in the mutant genotypes. (D–G) Confirmation of the expression levels of the indicated representative genes in replicate samples used in (A) by qPCR quantification. Transcript quantity is expressed relative to the control gene *EF1*.

To extract meaningful expression differences from our data, we then reasoned that wild-type and *hyh* seedlings do not display morphological differences in the test conditions. Thus, genes that are only different between *hyh* and wild-type, not between the double mutant and *hyh,* should not be responsible for the strong phenotypes observed in the double mutant. Rather, only genes that are consistently affected between the double mutant and both wild-type and *hyh* should be of interest (Figure 5). When applying these criteria, the expression levels of 627 genes (approximately 2.7% of all genes on the array) differ significantly (*p* < 0.05), applying a threshold of 2-fold (Table S1 and Figure 5A). Among them, 396 are underexpressed in *hy5 hyh* and 231 are overexpressed. Consistent with the milder phenotype of *hy5* single mutants compared to the double, only a subset of 263 (approximately 42%) of those genes is affected in *hy5* compared to wild-type (Figure 5A). One hundred fifty-one genes are underexpressed and 112 are overexpressed, and the direction of misregulation is generally similar to what is observed for those genes in the double mutant.

We searched our gene list for functionally defined genes that could explain the *hy5* and *hy5 hyh* phenotypes (Table 1). As expected, we found a number of light-regulated genes that have partly been reported to be under HY5 control, e.g., chalcone synthase *(CHS)* genes or chlorophyll a/b binding protein (CAB) genes and other components of the photosynthetic apparatus (16 genes in total) [4,15,20,21]. Other overrepresented, annotated gene classes that are misregulated in the double mutant include many transcription factors (57 genes) as well as ubiquitin ligase components (24 genes). Among the genes that stand out are *CIRCADIAN CLOCK ASSOCIATED* 1 *(CCA1)* [22], a circadian clock regulator previously suspected to be under *HY5* control [23], and *CUP-SHAPED COTYLEDON 1 (CUC1),* a gene involved in the organization of the apical embryo [24]. We confirmed the expression level trends of these particular genes in independent biological samples by qPCR experiments (Figure 5D and 5E). *CUC1* expression is hardly altered in *hy5,* but strongly so in the double mutant, consistent with the appearance of cotyledon fusions in the latter. Finally, another notable group of genes are genes that have been described to be involved in auxin-mediated transcriptional response (15 genes). We also confirmed the expression level trends of two genes from this group, *ARGOS* and *INDOLE ACETIC ACID (IAA) 19*, independently by qPCR (Figure 5F and 5G). No alterations were detected in the expression of genes that are implicated in auxin transport. Moreover, genes described to play a role in other hormone signaling pathways are conspicuously absent from our set, with the exception of four genes involved in gibberellic acid metabolism and one gene involved in ethylene biosynthesis (Table 1).

**Table 1:**
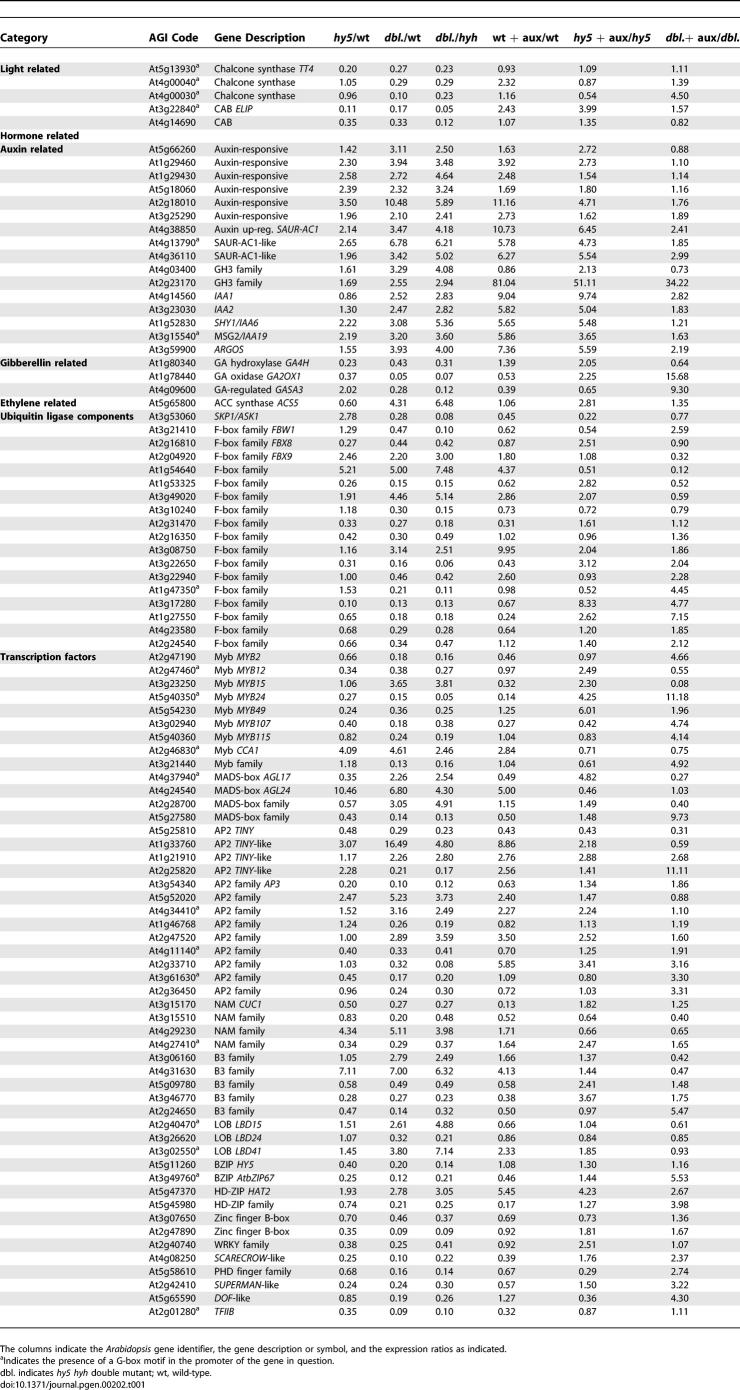
List of Selected Genes Consistently Misregulated in *hy5 hyh* Double Mutants, Ordered According to Functional Classification

In summary, as a general trend, the genes that are misregulated in *hy5* represent a subset of the genes misregulated in the double mutant. Moreover, the magnitude of misregulation is generally higher in the double mutant than in *hy5*.

### Constitutive Auxin-Induced Gene Expression in *hy5* and *hy5 hyh* Mutants

Because of the preponderance of auxin-related genes among the hormone-related ones, we investigated auxin-induced gene expression. Samples were grown in darkness for 3 d, exposed to light for 5 h, and then treated with externally applied auxin for 1 h. In the analysis of the respective microarrays, we concentrated on the set of 627 genes consistently affected in the double mutant. Of these, 246 (approximately 39%) were auxin-responsive in wild-type (threshold 2-fold, *p* < 0.05), with 129 genes being repressed and 117 genes being induced. A large proportion of these genes do not respond properly to auxin in *hy5* (166 genes, or approximately 66%) and the double mutant (199 genes, or approximately 82%) (Figure 5B and 5C and Table S1).

Of the 246 auxin-responsive genes of the wild-type, 112 do not respond to auxin treatment in *hy5 hyh* (i.e., approximately 46%; Figure 5B and 5C and Table S2). Most of those genes are highly misexpressed in untreated seedlings. Among the 66 genes that are induced by auxin in the wild-type, 62 are overexpressed in the double mutant, and of the 46 auxin-repressed genes, 39 are highly underexpressed. Thus, the data indicate that many auxin-responsive genes are constitutively expressed at auxin-induced or -repressed levels in *hy5 hyh* mutants.

Of the 112 genes that do not respond to auxin in the double mutant any longer, 58 are equally affected in *hy5* single mutants. The other 54 genes still react to auxin. In a small number of cases, opposite regulation in wild-type and *hy5* can be observed; however, as a general trend, with respect to auxin-regulated transcription, *hy5* mutants represent a mild version of *hy5 hyh* double mutants. This gradual increase in disturbance of auxin signaling is particularly evident in the expression of a number of well-characterized auxin-responsive genes. Some of these genes are involved in auxin signaling and have been described in the context of hypocotyl elongation and/or photomorphogenesis. For instance, *SHY1/IAA6* [10] is mildly overexpressed in *hy5* (approximately 2-fold), more affected in the double mutant (approximately 3-fold), still auxin inducible in *hy5*, but no longer auxin inducible in the double mutant (Table 1). The same is true for *IAA19/MSG2* [13]. In some cases, the gene is not affected in *hy5* but is in the double mutant, as seen for *IAA2*. A similar pattern can be observed for numerous other genes (e.g., Table 1). Moreover, in the set of 246 genes that are auxin responsive in the wild-type, many genes can be found whose auxin responsiveness is greatly diminished in *hy5* and/or *hy5 hyh* (Tables 1 and S1). Prominent examples include *IAA1* [25] (not affected in *hy5,* approximately 2.5-fold overexpressed in *hy5 hyh*, auxin responsiveness reduced to approximately 2.8-fold in *hy5 hyh* from approximately 9-fold in the wild-type) and *ARGOS* [26], a gene implicated in auxin-dependent lateral organ formation (not affected in *hy5,* approximately 4-fold overexpressed in *hy5 hyh,* auxin responsiveness reduced to approximately 5 and approximately 2-fold in *hy5* and *hy5 hyh,* respectively, down from approximately 7-fold in the wild-type). Similar patterns can be found for many genes annotated as auxin responsive, for instance, *SAUR-AC1* (approximately 2-fold overexpressed in *hy5,* approximately 3-fold in *hy5 hyh,* auxin responsiveness decreased from approximately 10-fold in wild-type to approximately 6-fold and approximately 2-fold in *hy5* and *hy5 hyh,* respectively). Finally, for a number of genes, inverse patterns can be observed (Tables 1 and S1); that is, they are repressed by auxin in the wild-type, but this repression is lost in *hy5 hyh* or *hy5,* because the respective genes are already underexpressed in those mutants.

In summary, the expression of many auxin-responsive genes is disturbed in *hy5* mutants and increasingly so in *hy5 hyh* double mutants. Comparison with the transcriptome data from untreated seedlings reveals that this lack of auxin response is generally reflecting a constitutive level of auxin-induced transcription in the mutants. This constitutive level is more severe in *hy5 hyh* mutants than in *hy5* mutants

### Polar Auxin Transport Is Altered in *hy5* and *hy5 hyh* Roots

**Figure 6:**
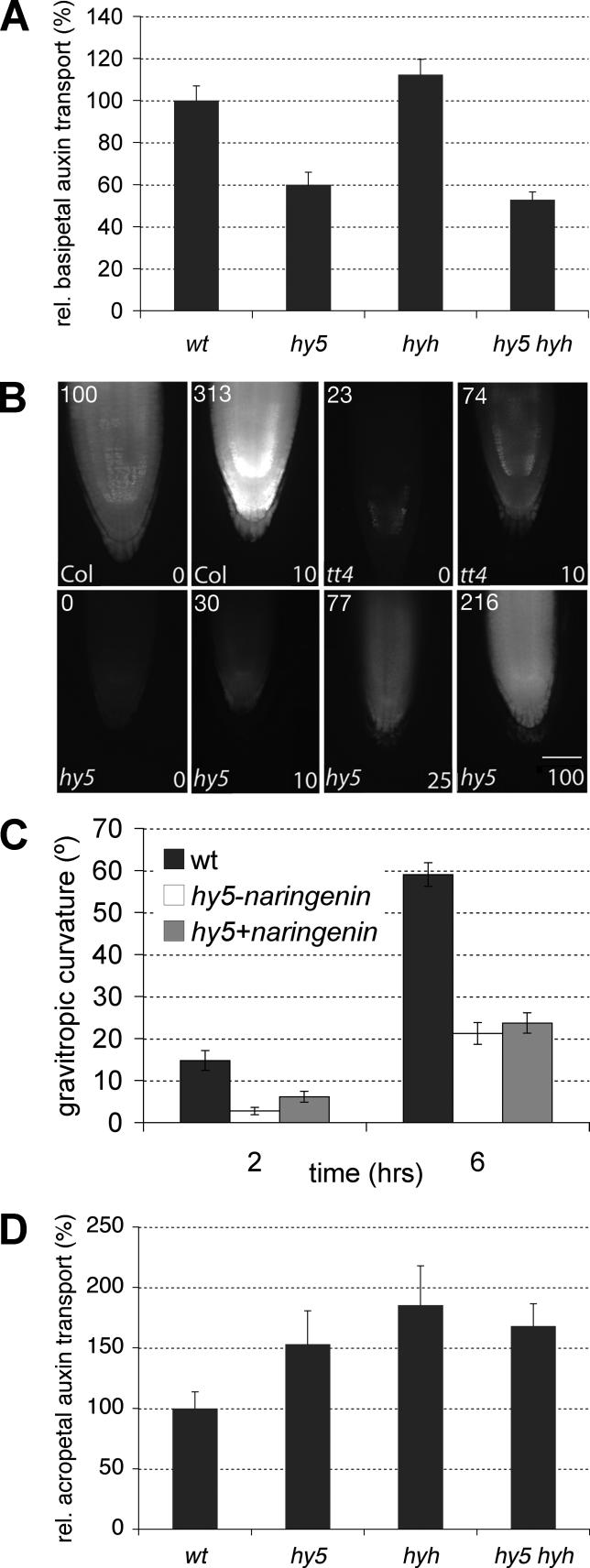
Flavonoid Content and Polar Auxin Transport Physiological analyses of wt, *hy5*, *hyh,* and *hy5 hyh* seedlings. (A) Relative basipetal auxin transport in the root tip. (B) Naringenin feeding restores flavonoid content in *hy5*. Fluorescent imaging of flavonoids in the primary root tip of *hy5* and the control genotypes wild-type (Col) and *tt4*. Naringenin concentrations are indicated in the lower right corner in μM. Values in the upper left corner represent quantification of flavonoid fluorescence relative (%) to wild-type. (C) Gravitropic response of the root tip (curvature) in wild-type, *hy5,* and *hy5* treated with 25 μM naringenin. Time points refer to time after change of the gravity vector by 90 degrees. (D) Relative acropetal auxin transport in the root. Error bars represent standard error of the mean.

Our microarray and phenotypic data implicate *HY5* and *HYH* primarily in auxin response at the transcription level. However, it is possible that the phenotypes might at least in part be due to altered auxin transport. For instance, root gravitropism requires correct auxin transport in the root tip [27]. Indeed, consistent with their agravitropism, basipetal auxin transport is reduced in *hy5* root tips (Figure 6A). In *hyh,* basipetal transport is normal, however, and the influence of *hyh* on this trait in the double mutant, if any, is marginal. Because flavonoids have been identified as regulators of basipetal auxin transport in the root [28] and because the expression of rate-limiting enzymes in flavonoid biosynthesis, notably CHS, is largely HY5 dependent [15,20,21], we investigated whether *hy5* root agravitropism is a secondary consequence of decreased flavonoid biosynthesis. To test this hypothesis, we fed *hy5* seedlings with naringenin, thus bypassing the requirement for CHS expression. A similar treatment rescues the flavonoid content and agravitropism of *transparent testa 4 (tt4),* a null mutant in the principal *CHS* gene [28]. Sufficient levels of naringenin indeed also restore flavonoid content in the *hy5* mutant (Figure 6B); however, agravitropism (Figure 6C) or other *hy5* root phenotypes are not rescued.

Opposite to basipetal transport, acropetal auxin transport in *hy5* roots is enhanced rather than reduced (Figure 6D). This is also the case in *hyh* and in double mutant roots; however, no enhancement of this trait is observed in the latter. Thus, both basipetal and acropetal auxin transport are altered in *hy5* and *hy5 hyh* roots.

## Discussion

### The Phenotypes of *hy5*, *hyh,* and *hy5 hyh* Mutants: A Paradoxical Situation

Our analysis of the developmental roles of *HY5* and *HYH* revealed two paradoxes. First, *hy5* null mutants have dramatic seedling phenotypes, while no morphological phenotype is apparent in *hyh* null mutants, except slightly defective inhibition of hypocotyl elongation specifically in blue light [4]. In light of our expression analyses, this is surprising. Both genes are expressed in nearly identical patterns, and in light-grown seedlings *HYH* is more abundant than *HY5,* although it nevertheless is apparently the dispensable one of the two genes. Finally, the HY5 and HYH proteins are functionally equivalent, because ectopic overexpression of either gene rescues *hy5* phenotypes equally well. Importantly, neither gene is required for the expression of the other (R. Sibout, unpublished data). However, it has been described that HYH protein steady state levels are decreased in *hy5* [4]. This reduction might be functionally more significant than initially suspected, because of the increased stability of the altHYH protein, which is likely due to the lack of the COP1 interaction domain. Thus, conceptually, *hy5* could be considered a weak *hy5 hyh* double mutant.

The biological significance of the alternative *HYH* transcript remains to be determined. Although *altHYH* is expressed at relatively low levels in darkness when compared to *HY5*, due to the increased stability of altHYH protein, the difference in protein activity is likely less dramatic. Thus, altHYH could have an important role in kick-starting gene expression upon light stimulus and in sustaining light-regulated gene expression once the initial burst of light-induced *HY5* transcription is dropping to steady state levels. It is also conceivable that altHYH could play a role in the anticipation of dawn. Although our experiments do not differentiate between diurnal and circadian regulation of *HYH,* a direct link to the circadian clock is suggested by our finding that its central component *CCA1* appears to be under *HY5/HYH* control.

The second paradox is the discrepancy between the *hy5* and double mutant root phenotypes. While lateral root emergence and growth is enhanced in *hy5* mutants, this phenotype is suppressed in the double mutants. Moreover, overall root system growth is even diminished in *hy5 hyh*. This situation correlates with a gradual increase in constitutive auxin signaling in the *hy5 hyh* versus the *hy5* mutant background, as indicated by transcriptome analyses and by the occurrence of novel, auxin-related phenotypes in *hy5 hyh* double mutant shoots.

### A General Role of *HY5* and *HYH* in Auxin Signaling

Initially, *HY5* had been suspected to play a role in auxin signaling because of the branching phenotype and agravitropism of *hy5* roots [3]. The double mutant, however, implicates *HY5* and *HYH* in auxin signaling in a much wider sense, because the observed novel phenotypes are hallmarks of strongly impaired auxin signaling. The appearance of fused cotyledons in *hy5 hyh* is an especially specific indicator. Because the cotyledons are formed in the embryo, this phenotype reveals a role for *HY5* and *HYH* in embryogenesis. Cotyledon fusions are observed in a number of auxin signaling embryogenesis mutants, e.g., *bodenlos (bdl/iaa12)* [29] or *monopteros* (*mp*) [30]. Interestingly, this phenotype is usually not fully penetrant and represents the extreme of a phenotypic range. The *hy5 hyh* double mutant is similar in this respect: fused cotyledons are only observed in approximately 15% of seedlings. However, the cotyledons of all *hy5 hyh* seedlings have an altered vasculature, which is also a characteristic of mutants altered in auxin signaling or transport, e.g., *mp*. The correct separation of cotyledons in embryogenesis requires the three partially redundant *CUC* genes [24,31]. Among them, *CUC2* acts downstream of *MP* and the auxin transport regulator *PIN-FORMED 1* (*PIN1*) [32]. By contrast, *MP* and *PIN1* do not control embryonic expression of *CUC1*. Our microarray experiments indicate that the expression level of this gene is strongly altered in *hy5 hyh* double mutants. Therefore, *HY5/HYH* control of *CUC1* might act as a parallel input to provide maximal *CUC* activity in embryogenesis. In summary, the strong auxin-related phenotypes of *hy5 hyh* double mutants, combined with the results from our microarray analyses, suggest that *HY5* and *HYH* have a general role in auxin signaling, from embryogenesis on throughout seedling development.

### A Complementary Strategy for Transcriptome Analysis of *hy5/hyh* Mutants

In this study, we minimized secondary expression changes in transcriptome analysis by choosing a developmental stage at which mutants and wild-type display minimal morphological differences, but the genes in question are known to be nevertheless active. Our approach revealed that about 2.7% of tested genes are consistently misexpressed in *hy5 hyh,* a number that is in the range of a previous experiment, although array types and biological material used are not comparable [4]. One disadvantage of our strategy is that genes primarily affected at later stages of development are missed. For instance, the dark-grown seedlings used in this study had not yet developed lateral root primordia. This is presumably why, for instance, *SOLITARY ROOT* (*SLR/IAA14*), previously found to be misexpressed in *hy5* [15] and consistent with its role in lateral root emergence [33], is not affected in this study.

The total number of genes misexpressed in *hy5 hyh* is 627, with approximately two thirds underexpressed and one third overexpressed. Notably, HY5 and HYH do not possess transcription activation or repression domains and likely act in concert with other factors [4]. Therefore, it is conceivable that they could repress the expression of some genes but activate the expression of others. It seems unlikely that all 627 genes are direct targets of HY5 and/or HYH. In general, the promoters of direct target genes would be expected to contain a HY5/HYH binding site, the G-box motif. One or more G-box motifs can be found in the 5′ 1,000-bp promoter regions of 97 genes (C. S. Hardtke, unpublished data). These include, for instance, *CCA1, CHS,* and *CAB* genes and a number of transcription factor genes (Table 1). Interestingly, among the auxin-responsive genes, G-box motifs are not more frequent than in the complete set of 627 genes (approximately 15% to 16%). Nevertheless, the proportion of auxin-responsive genes among the 627 genes affected in the double mutant (approximately 39%) significantly exceeds the proportion of auxin-responsive genes in the wild-type (approximately 16%). Thus, in line with the phenotypic analysis of *hy5 hyh* double mutants, *HY5* and *HYH* clearly modulate auxin-regulated gene expression but most likely by controlling a few central regulators of auxin response. Among the functionally defined auxin signaling genes affected in the double mutant, only *MSG2/IAA19* contains a G-box motif. Interestingly, as in *AXR2/IAA7* and *SLR/IAA14* [15], it is located close to the transcription initiation site. Consistent with *hy5* and *hy5 hyh* phenotypes, *MSG2/IAA19* has been implicated in hypocotyl growth responses and lateral root formation [13]. Moreover, *MSG2/IAA19* is a highly light-responsive gene that is expressed at very high levels in darkness and repressed upon light stimulus, a feature it shares with the equally affected *SHY1/IAA6* gene [34]. Thus, *MSG2/IAA19* could represent one of the central auxin signaling components that directly link light- and auxin-induced gene expression.

### Auxin Signaling versus Auxin Transport

While the transcriptome analyses demonstrate a perturbation of auxin signaling, it also appears possible that the *hy5* and double mutant phenotypes rather result from altered auxin transport. For example, the reduced basipetal auxin transport is consistent with the agravitropism of *hy5* roots and the increased acropetal transport with enhanced lateral root emergence. In general, however, it is difficult to determine whether the primary defect is in auxin transport or signaling, because of the inherent feedback connections between the two processes [35]. Several observations favor the interpretation that altered auxin transport in *hy5 hyh* mutants is a secondary consequence of altered auxin signaling. For instance, root agravitropism and lateral root emergence defects of the *axr4* auxin transport mutant can be rescued by application of the lipid-soluble auxin analog NAA [36,37]. This is, however, not the case for *hy5* or the double mutant (C. S. Hardtke, unpublished data). Also, our naringenin feeding experiments exclude the possibility that auxin transport is altered because of reduced flavonoid biosynthesis, since naringenin treatment did not restore gravitropism in *hy5* roots. In contrast, growth of flavonoid-deficient and agravitropic *tt4* mutant seedlings on naringenin restores gravity response [28]. Moreover, acropetal auxin transport is significantly enhanced to the same degree in *hy5, hyh,* and the double mutants, but their root branching phenotypes are very different. Finally, HY5 and HYH are transcription factors, but genes directly implicated in auxin transport are conspicuously absent from the set of genes with altered expression levels in the mutants. Therefore, the combined evidence suggests that *hy5* and *hy5 hyh* mutants are mainly defective in the perception or interpretation of auxin stimulus (i.e., auxin signaling), and alterations in auxin transport are likely a secondary consequence of feedback regulation.

### Auxin-Related Phenotypes in *hy5* and *hy5 hyh* Mutants: A Quantitative Affair?

Our transcriptome analyses and the novel *hy5 hyh* shoot phenotypes support the hypothesis that auxin signaling is constitutively disturbed in *hy5* and the double mutants. The transcriptome analyses also suggest that the molecular defects in *hy5* and *hy5 hyh* are largely similar although quantitatively different. The number of misregulated auxin-responsive genes is higher in the double mutant than in *hy5*. Moreover, in the case of genes that are affected in both genotypes, the magnitude of misregulation is always considerably higher in the double mutant. Finally, the auxin-induction experiments clearly indicate that the vast majority of auxin-responsive genes that are affected in the double mutant are expressed at constitutively auxin-induced or -repressed levels. Therefore, in the microarray analyses, *hy5 hyh* mutants largely mimic auxin-treated wild-type, suggesting that auxin signaling is constitutively elevated. This is also true for *hy5,* albeit at a lower quantitative level.

**Figure 7:**
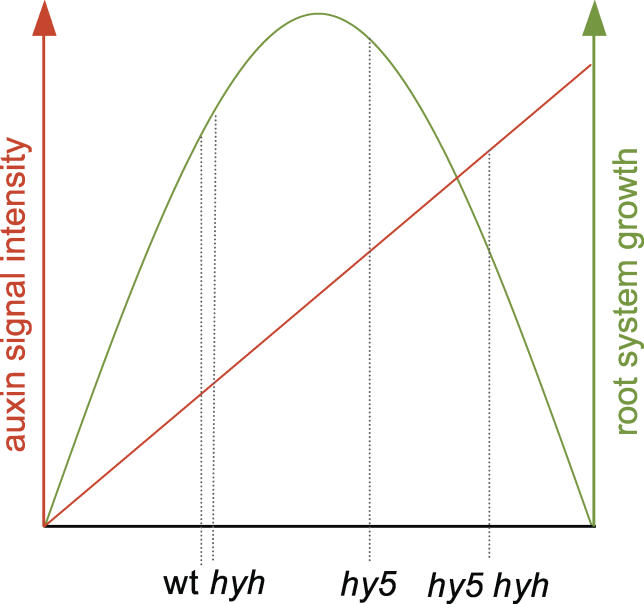
Correlation of Root System Growth and Auxin Signaling Graphical presentation of a model explaining the opposite root system phenotypes of *hy5* and *hy5 hyh* mutants. The strength of (constitutive) auxin signaling in the respective genotype determines the extent of root system growth. Root system growth is maximal at a certain optimum auxin signaling level but decreases if this level is either lower or higher. This assumption is supported by the phenotypic response of wild-type seedling root systems to the external application of increasing amounts of auxin: whereas low auxin concentrations promote root system growth, high concentrations inhibit growth.

The phenotypic progression from wild-type to *hy5* to the double mutant correlates with quantitatively ever-increasing disturbance of auxin-responsive gene expression, reflecting ever-increasing constitutive auxin signaling. Thus, it appears that the smaller increase in constitutive auxin signaling in *hy5* accelerates root system growth, while a further increase beyond a critical threshold, a situation created in the double mutant, suppresses growth (Figure 7).

### Conclusions

In summary, we therefore conclude that the disparate root system growth phenotypes of *hy5* and the double mutant reflect a morphological response to a quantitative gradient in the same molecular process, i.e., a gradual increase in constitutive auxin signaling. Finally, because practically all the classic auxin responses, from tropisms to lateral organ formation to cell elongation and proliferation, are impaired in *hy5 hyh* double mutant seedlings, we conclude that *HY5* and *HYH* are important general negative regulators of the auxin signaling pathway, modulating its amplitude from embryogenesis throughout seedling development.

## Materials and Methods

Phenotypic differences in measurements highlighted in the Results section are all statistically significant at *p* = 0.01 based on Student's *t*-test.

### Plant material and tissue culture.

Seedlings were grown at 22 °C under constant illumination on culture medium (0.5× MS salts, 0.5 g/L MES, 1% sucrose, 0.9% agar [pH 5.8]) unless otherwise stated. Light intensity was approximately 100 μE. In all assays, the *hy5-KS50* null allele, the *hyh* T-DNA insertion null allele, and the respective double mutant combination were analyzed in comparison to their wild-type background, Ws*.* In the auxin transport assays and microarray analyses, the *hy5–215* null allele was compared to its background parent, Col.

### Confocal analysis of roots.

To determine mature cell size and meristem size in the primary roots of the different genotypes, 7-d-old propidium iodide–stained seedlings were observed by confocal microscopy. For mature cell size, cortex cells were measured; for meristem size, proliferating cells in the meristematic zone were counted as described [38].

### Physiological assays and phenotyping.

Morphological phenotypes were determined in tissue culture for at least 100 seedlings. Auxin transport assays, naringenin feeding experiments, and gravitropism assays were performed as described [28] with a minimum of 20 scored seedlings per treatment and genotype. For adventitious root induction, 12 or more 5-d-old etiolated plants were transferred into light and adventitious root emergence was monitored after 5 and 12 d.

### Creation and analysis of transgenic plants.

Plasmids were created by amplification of *HY5* or *HYH* promoter fragments (from 5′ to the ATG up to the neighboring gene) or ORFs from genomic or cDNA templates, respectively, with Pfu polymerase (Fermentas, http://www.fermentas.com), followed by cloning into binary vector pTCSH1 [19] or its derivatives. Binary constructs were verified by sequencing and transformed into *hy5–215* single mutant or *hy5-KS50 hyh* double mutant plants via floral dip using Agrobacterium. Transgenic lines were selected by screening seed progeny for glufosinate ammonium or hygromycin resistance on medium containing 0.3% sucrose. Independent lines segregating single locus insertions and stably expressing the transgenes in the T3 generation were chosen for analysis.

### Western analyses.

Light-to-dark shift experiments and detection of HYH proteins using anti-HYH antibody was performed as described [4].

### Quantitative real-time RT-PCR.

Total RNA was isolated from seedlings, roots, or shoots (20 individuals) using the RNeasy kit with DNase step (Qiagen, http://www.qiagen.com). Reverse transcription (Promega, http://www.promega.com) was carried out according to the manufacturer's instructions using 1.5 μg of total RNA and oligo-dT primers. Samples were treated simultaneously. qPCR analyses were carried out on a Stratagene Mx3000P apparatus (http://www.stratagene.com) using SYBR green dye technology (http://www.bio-rad.com). PCR amplifications of 45 cycles were done in two-step reactions, with a denaturation of 10 s at 94 °C and an elongation of 2 min at 68 °C. Gel electrophoresis was systemically done to verify amplicons. Absolute quantity of transcripts was calculated using DNA standard curves [39]. Results are presented in-fold change or absolute values, standardized in relation to the *EF1* housekeeping gene. All qPCR data represent the average of two *(CCA1, CUC1, ARGOS, IAA19)* or three *(HY5, HYH)* independent biological and technical replicates. Primers used for *CCA1* were GCCGCAGTAGAATCAGCTCCAATATAA and GAAGCATCTAATCCGATTCCAAGAA. Primers used for *CUC1* were GCACGTGTCCTGTTTCTCCAATAA and ATCTGTCCCGATGATCCCAAA. Primers used for *ARGOS* were CGGAGTTTCTCGGCGCAGAAA and CAATGGGAACCAATAGCAGCATAAA. Primers used for *IAA19* were GTCATGCAAGAGGTTGAGAATAA and AACTCAACACTCAAGAAACAAGTA. Primers used for *altHYH* were CCCACAAGAAGCACAAAACTGCTGGAT and CACGGCGGCGTTTAGCTGTAGAGA. Primers used for *flHYH* were CCCACAAGAAGCACAAAACTGAGGAAA and CTTCCACGGCGGCGTTTAGCTGTAGAGA. Primers used for *HY5* were CCATCAAGCAGCGAGAGGTCATCAA and CGCCGATCCAGATTCTCTACCGGAA. Primers used for *EF1* were GGTCACCAAGGCTGCAGTGAAGAA and GCTCAAACGCCATCAAAGTTTTAAGAA.

### Microarray analyses.

For microarray analysis, 20 or more 3-d-old etiolated seedlings grown on sugar-free medium were transferred into liquid medium and into the light. Plants were treated or mock-treated with 10 μM IAA after 5 h of light induction and harvested 1 h later. Total RNA was isolated using RNeasy kits (Qiagen) according to the manufacturer's instructions. Labeling and hybridization of ATH1 DNA arrays (22k) (Affymetrix) was performed according to the manufacturer's instructions (http://www.affymetrix.com/support/technicalmanual/expression_manual.affx). Intensity values were normalized with the MAS 5.0 method. Data were analyzed using the RACE software and the Bayes test for statistical significance [40]. Variations in expression level were considered significant only if the fold change was greater than 2 with a probability lesser than or equal to 0.05. Two biological replicates were performed for all samples.

## Supporting Information

Table S1 List of All Genes Consistently Misregulated in *hy5 hyh* Double Mutants The columns indicate the Affymetrix probe identifier, the *Arabidopsis* gene identifier, the gene description, and the expression ratios as indicated. dbl. indicates *hy5 hyh* double mutant.
(195 KB XLS)

Table S2 List of All Genes that Were Auxin Responsive in the Wild-Type and Their Response in the Mutant Genotypes The columns indicate the Affymetrix probe identifier, the Arabidopsis gene identifier, the gene description, and the expression ratios as indicated. dbl. indicates *hy5 hyh* double mutant.
(88 KB XLS)

### Accession Number

The raw data are available from the ArrayExpress database (http://www.ebi.ac.uk/arrayexpress) under accession number E-MEXP-715.

## Abbreviations

*altHYH*: alternative *HYH* transcript
CAB: chlorophyll a/b binding protein
CHS: chalcone synthase
COP1: CONSTITUTIVE PHOTOMORPHOGENIC 1
*flHYH*: full-length *HYH*
HY5: LONG HYPOCOTYL 5
HYH: HY5 HOMOLOG
qPCR: quantitative real-time RT-PCR
*tt4*: *transparent testa 4*
